# Processing of translation units by student and semi-professional translators in translating texts with different levels of translation difficulty

**DOI:** 10.1371/journal.pone.0320809

**Published:** 2025-04-08

**Authors:** Fuxiang Wang, Qingli Xu

**Affiliations:** 1 Center for College English Education Research, Qufu Normal University, Qufu, Shandong, Peoples’ Republic of China; 2 College of Foreign Languages, Qufu Normal University, Qufu, Shandong, Peoples’ Republic of China; Sreenidhi Institute of Science and Technology, INDIA

## Abstract

This study investigates the impact of translation difficulty and translation experience on the processing of translation units (TUs) based on keylogging data. TUs are categorized into two types: production TUs (PTUs), which have linguistic equivalents in the source text (ST), and non-production TUs (N-PTUs), which lack such equivalents. N-PTUs typically include combinations of letters, morphemes, or non-text production keystrokes, such as backspaces, deletions, spaces, cursor movements, carriage returns, and mouse navigations. Data were collected from 55 fourth-year English major students and 35 semi-professional translators as they translated two STs of differing difficulty. The findings reveal no significant differences in the number of PTUs or the average size of PTUs (APTU size) between students and semi-professional translators. However, ST translation difficulty significantly influenced TU processing. For the more difficult text, the APTU size decreased notably, the number of 1–6-word PTUs increased substantially, and the frequency of ≥ 7-word PTUs remained relatively stable. Moreover, when translating the difficult text, semi-professional translators produced smaller APTU sizes, a greater number of N-PTUs, and a higher overall average number of PTUs compared to their performance on the relatively easy text. These results highlight the interplay between cognitive effort, ST translation difficulty, and translation experience in TU processing and have some implications for designing translation courses in academic contexts and developing professional translator training programs.

## Introduction

Generally, in translating a translator needs to sever the source text (ST) into easily manageable segments to ensure the flow of translation. Such segments are referred to as translation units (TUs). TU has been a longstanding topic of debate in translation studies [1,2]. TUs have been examined from both product-oriented and process-oriented perspectives. From a product-oriented perspective, TUs are perceived more statically as properties observable in the translation product, that is, correspondences in the pair of texts as a result of the translation process [3], and the size of TU is taken to range from the morpheme to discourse [4–9]. From a process-oriented perspective, TUs are taken as ST segments to which the translator’s focus of attention is directed [10], i.e., the cognitive units processed in the translator’s mind while translating.

Although these ST segments are not directly perceptible, it is assumed that by analyzing the continuous text production that follows the translator’s focus of attention, a TU can be momentarily captured as a segment of text production between pauses of a certain length. Thus, a TU can be concretized as the flow of continuous target text (TT) production separated by pauses during the translation process as registered by think aloud protocols (TAPs), key-logging and/or eye-tracking software [3,11–14], and the size of TU is computed as the number of words between two pauses of a certain length [2]. In the keylogging data, TUs are categorized into two types: production TUs (PTUs) and non-production TUs (N-PTUs). PTUs refer to TUs that have a more or less equivalent linguistic entity in the ST, whereas N-PTUs consist of TUs that lack such equivalents. N-PTUs are generally formed by combinations of letters, morphemes, or non-text production keystrokes, including backspaces, deletions, spaces, cursor movements, carriage returns, and mouse navigations.

This study aims to calculate and compare the number and size of the TUs produced by 55 fourth year English major students and 35 Master of Translation and Interpreting (MTI) students in a Chinese university in the translation of two relatively easy and difficult texts in the key-logging software Translog II [15,16]. It investigates whether ST translation difficulty and translation experience significantly affect the number and size of PTUs and the number of N-PTUs. Additionally, it examines whether an interaction effect exists between ST difficulty and translation experience with respect to the number and size of PTUs and the number of N-PTUs.

## Literature review

The size of TU has been a central interest in process-oriented translation studies [17]. Early studies, drawing on think-aloud protocols (TAPs) data, identified the prototypical TU as a group or a clause typically comprising two to six words [11,12,18,19]. These studies further examined how size of TU is related to translation experience and ST difficulty.

### Relationship between production of TU and translation experience

The size of TU has been widely reported to correlate strongly with translation experience, with professional translators typically processing longer or larger TUs compared to novices. For instance, Gerloff [20] found that TUs produced by student translators are primarily morphemes and words. Similarly, Lörscher [21–23] demonstrated that translation learners tend to focus on single words as TUs, whereas experienced translators are more likely to isolate and process units of meaning, which are typically realized as phrases, clauses, or sentences. Supporting this, Séguinot [24] and Barbosa and Neiva [25] reported that professional translators predominantly employ phrases, clauses, and sentences as TUs, which are significantly larger than the TUs adopted by student translators. Jääskeläinen and Tirkkonen-Condit [26] also observed that professional translators process larger TUs compared to non-professionals. Dragsted [13], based on keylogging and TAPs data, found that professional translators, on average, handle longer segments than students, though both groups frequently process three-word TUs. Additionally, Englund-Dimitrova [27] identified a group-level correlation between translation experience and the ability to plan and write larger chunks of TT as cohesive segments without interruptions. More experienced translators divided their translation process into fewer TUs, each encompassing a greater number of characters. Jakobsen [28] also found that expert translators worked in longer segments than translation students. Keylogging and eye-tracking studies, such as those conducted by Carl and Kay [3] and Barbosa and Neiva [25], further corroborated these findings, revealing that professional translators tend to process longer stretches of text compared to translation students.

However, not all studies have found a clear relationship between translation experience and size of TU. For example, Kiraly [19], in a TAPs study, reported that both professional translators and language students predominantly process words and short word strings as TUs, with no significant differences in size of TU. Kiraly’s findings indicated only a marginal difference in the average length of word strings, with professionals slightly exceeding the overall average (3.9 words per string) and students slightly below it (3.6 words per string). Similarly, the non-significant difference between professional translators and student translators in size of TU was also observed in Dragsted [13]. Based on the keylogging and TAPs data, the average size of TU for the professional translators was found to be typically around or above 4 words per unit (4.4 words), whereas for the student translators, the figure was around or below 3.5 words (3.6 words). But when the exceptionally long segments were deducted from the average segment size, there is only slight, non-significant difference between the two groups. In general, the two groups had a tendency to process segments of the same size, i.e., the range of the most frequent segment size was the same for both groups, viz., two to four words.

### Relationship between ST translation difficulty and production of TUs

The size of TU has also been found to be related to ST translation difficulty. For instance, Dragsted [13,29] reported a highly significant difference in the average size of TU in translating two easy and difficult texts, alongside a marginally significant participant/text interaction effect. The simple effects tests were conducted and the results revealed a relatively larger difference in size of TU between professional and student translators for the easy text compared to the difficult text. In other words, the difference in size of TU observed in the easy text was partially neutralized in the difficult text. Similar findings were reported by Wang [30], who analyzed keylogging and TAPs data collected from 12 foreign language students and 12 semi-professional translators tasked with translating two relatively easy and difficult Chinese texts into English. The students’ average size of TU for the easy and difficult texts was 4.48 and 4.38 words respectively, while for the semi-professionals, it was 5.36 and 4.18 words respectively. These results indicate that both ST translation difficulty and translation experience influenced size of TU to varying extents. Repeated measures ANOVA results showed that the average size of TU decreased when translating the difficult text. Moreover, the difference in size of TU between student and semi-professional translators for the easy text was neutralized in the difficult text, with semi-professionals adopting characteristics akin to students. Extending this line of research, Wang [2] conducted a keylogging study in which 38 students translated two relatively easy and difficult texts. Unlike earlier findings, this study observed no significant reduction in average size of TU for the difficult text.

In summary, there exist notable disparities in the findings and methodologies employed in the afore-mentioned studies. First, the results reported by Dragsted [13], Kiraly [19], Englund-Dimitrova [27], and Wang [30] contradict other studies, which suggest that professional translators typically process larger TUs compared to students. These inconsistencies necessitate further clarification and investigation. Second, there is inconsistency in the reported relationship between size of TU and ST translation difficulty. While Dragsted [13,29] and Wang [30] identified a significant effect of ST difficulty on size of TU, Wang [2] found no such effect. Thus, it remains unclear whether ST difficulty significantly influences size of TU. Third, most studies have focused solely on student and professional translators, overlooking semi-professional translators. This gap limits our ability to describe translators’ cognitive development with finer granularity. Finally, the small sample sizes in these studies, as shown in Table 1, increase the likelihood of false positives or the omission of significant differences. To address these issues, we conducted an experiment with a larger sample, including both student and semi-professional translators, to examine how translation experience and ST translation difficulty influence TU processing, particularly in terms of number and size of TUs.

**Table 1:** The samples in some studies.

Researcher	Gerloff	Krings	Lörscher	Kiraly	Séguinot	Barbosa & Neiva	Dragsted	Jakobsen	Carl & Kay
Year	1986	1986	1986	1995	1996	2003	2004	2005	2011
Sample size	5Ss^1^	8Ss	15Ss	8Ps^2^ &10Ss	2Ps	Several Ss & Ps	6Ps & 6Ss	5Ps & 4Ss	12Ps & 12Ss

^1^Ss = students;

^2^Ps = professionals.

## Methods

### Research questions

This study aims to explore the following questions:

1. Are there differences in the number and size of TUs between student translators and semi-professional translators?
2. Do the number and size of TUs differ between an easy text and a difficult text?
3. Is there an interaction effect between translation experience and ST translation difficulty on the number and size of TUs?

### Participants

Two groups of participants were randomly selected to take part in this empirical study. The first group comprised 55 senior (fourth year undergraduate) English majors (47 female) from a university in Shandong Province, China, while the second group included 35 second-year MTI (Master of Translation and Interpreting) students (29 female) from the same institution. All participants were native Chinese speakers who had learned English as a foreign language and had no prior experience studying in English-speaking countries. All participants reported passing TEM 4, a national English proficiency test specifically designed for second-year English majors. Moreover, the MTI students also reported passing TEM 8, the advanced national English proficiency test for final-year English majors, which the senior English majors had not yet attempted. Both TEM 4 and TEM 8 assess students’ comprehensive English skills, including listening, reading, writing, and translation.

The senior students had completed two semesters of coursework in Chinese-English translation but reported no professional translation experience. In contrast, the MTI students had completed a year of coursework in Chinese-English translation as well as a second year of professional practice, during which they translated at least 100,000 characters or words between Chinese and English; they all also reported having passed the Level II of CATTI (The China Accreditation Test for Translators and Interpreters), the intermediate-level certification for translation professionals. Following the classifications used in studies such as Englund-Dimotrova [27], Jääskeläinen [31], Jakobsen [32], and Zheng & Tan [33], the senior students were categorized as novice translators, while the MTI students were considered semi-professionals. While neither group was entirely homogeneous in terms of translation experience, the MTI students demonstrably had more extensive experience than their senior student counterparts. None of the participants had prior experience translating legal texts.

The study received ethical approval from the Biomedical Ethics Committee of Qufu Normal University (#2023152) before participant recruitment and data collection. Recruitment was conducted between February 1, 2023, and March 6, 2023. Written informed consent was obtained from all participants, who were each compensated with an honorarium of 60 yuan for their time and effort.

### Participants’ typing speed

To account for the potential influence of typing speed on keylogging data, all participants were instructed to copy a 100-word English passage using Translog II. This step was taken to determine whether there was a significant difference in typing speed between the senior students and the MTI students. The copying durations were recorded, and a two-tailed independent samples t-test was conducted. The results indicated no significant difference between the two groups (t =  0.301, p =  0.869 >  0.05).

### STs and determination of their level of translation difficulty

Both groups of participants were tasked with translating two different texts from Chinese (their mother tongue) into English (their foreign language). The first text, an allegorical story for children titled *The Farmer Who Waited by the Stump for Another Hare to Run into* (Text A), features concise and vivid language, avoiding complex or lengthy descriptions, and is generally considered easier to translate. The second text, an excerpt from an apartment lease contract (Text B), was selected for its potential to introduce terminological challenges for both groups. Both texts were authentic and carefully adapted to the same length (212 Chinese characters). Text B was deliberately chosen to ensure, or at least increase the likelihood, that all participants, including the semi-professional group, would encounter certain translation difficulties.

After completing the translation tasks, participants were asked to rate the difficulty of translating each text using a 5-point Likert scale. A two-tailed paired samples t-test results revealed that Text B was significantly more challenging than Text A (t =  47.589, p =  0.000 <  0.05).

### The keylogging software Translog II

Translog II, developed by Michael Carl in 2012 [15], as an extension and enhancement of the original Translog introduced in 1995 by Jakobsen and Schou [16], is a sophisticated keylogging software widely recognized in translation studies for analyzing text production processes. It consists of two main components: Translog-supervisor (Supervisor) and Translog-user (User). The Supervisor enables researchers to design tasks, configure settings, and analyze collected data through functionalities such as replaying, linear representation, and detailed statistical analysis. Meanwhile, the User interface provides participants with the ST and a dedicated workspace for entering translations, facilitating a seamless translation experience within the software environment.

Specifically designed for translation process research, Translog II has become an indispensable tool for capturing, analyzing, and visualizing all keystrokes and mouse movements made by translators during the translation process. Its advanced features provide researchers with detailed insights into the cognitive and behavioral patterns underlying translation activities. Using the linear representation of keylogging data, TUs can be identified based on specified pause thresholds. Researchers can further calculate the number of TUs, including PTUs and N-PTUs, as well as the size of PTUs, offering a robust framework for investigating translation processes.

### Data collection

Key-logging data were collected using Translog II in a university language laboratory. Before beginning the translation task, participants completed a sample Chinese-to-English translation exercise to familiarize themselves with the computer keyboard and the Translog II interface. They were then provided with a written translation brief, which specified the target audience for each text. For Text A, the target readers were children of American teachers at the university who were attending primary school, while for Text B, the target readers were American students coming to the university to learn Chinese. Participants were instructed to produce translations that aligned with their usual standards of translation quality. There was no time limit for the task, and participants were allowed to use the Youdao online English–Chinese bilingual dictionary, though access to other websites was prohibited. To mitigate potential acclimatization effects, the two texts were presented in a semi-randomized sequence.

### Setting the pause threshold and calculating size of TU

Pauses in text production are behavioral indicators of the cognitive processes underlying shifts in attentional state [34]. As such, pauses can be used to identify boundaries between TUs [35]. However, there remains a lack of consensus among researchers regarding the minimum pause duration that should qualify as a marker of TU boundaries [36], with pause thresholds often chosen to suit specific research objectives. A pause threshold of 2.4 seconds has been widely adopted in studies by Alves et al. [37], Alves and Gonçalves [38], De Lima Fonseca [39], Jakobsen [28], Sekino [40], and Wang [2], as this parameter has been shown to yield more consistent results for TU segmentation. To ensure consistency and comparability, the present study also applied a pause threshold of 2.4 seconds.

Based on this threshold, and following the operationalization proposed by Englund-Dimitrova [27], a TU in this study was defined as a segment of the TT bounded by two pauses of ≥ 2.4 seconds during the TT production process, as recorded by Translog II. This includes deletions, additions, and other potential online modifications. In the linear representation of the Translog data, TUs can generally be classified into two types: production TUs (PTUs) and non-production TUs (N-PTUs). PTUs are segments of the TT that have a linguistic equivalent in the ST and form part of the TT, while N-PTUs lack such equivalents and are typically composed of letter combinations, morphemes, or non-text production keystrokes, such as backspaces, deletions, spaces, cursor movements, carriage returns, and mouse navigations.

In this study, the size of a PTU was calculated as the number of words that it contained. PTUs were categorized into 10 levels, ranging from one word to ≥ 10 words [2]. Namely, if a PTU consists of only one word, it is a 1-word PTU; if it consists of five words, it is a 5-word PTU. And so forth. A participant’s average size of PTU (APTU size) for a given text was determined by dividing the total number of words produced in the TT by the total number of PTUs identified during the translation process.

### Experimental design and statistical analysis model

This study employed an experimental research design with two dimensions: translation experience and ST translation difficulty. A 2 × 2 mixed design was adopted, where the factors were translation experience (student vs. semi-professional) and ST translation difficulty (easy vs. difficult). Translation experience served as the between-subject variable, and ST translation difficulty was the within-subject variable. The independent variables were translation experience and ST translation difficulty, and the dependent variables were the number and size of TUs. Statistical analyses were primarily conducted using repeated measures ANOVA in SPSS 29.0 to examine whether there were significant main effects of translation experience and ST translation difficulty, as well as to determine whether an interaction effect existed between the two factors.

## Results

### Number and size of TUs in the translation of Text A and Text B

This section presents the variations in the overall average of PTUs, the average number of PTUs at each level, and the APTU sizes in the translation of Text A and Text B.

#### Comparison of average number of production words, PTUs and N-PTUs.

The average number of TT production words, PTUs, and N-PTUs produced by all participants in the translation of Texts A and B was calculated, as shown in Table 2. The results indicated that, on average, participants used approximately 54 more production words, 19 more PTUs, and 9 more N-PTUs when translating Text B compared to Text A. These differences were further validated by the results of repeated measures ANOVA, which demonstrated a significant main effect of ST translation difficulty. Specifically, significant differences were observed between the two texts in the average number of production words (F(1, 88) =  432.77, p < .000), PTUs (F(1, 88) =  262.53, p < .000), and N-PTUs (F(1, 88) =  56.281, p < .000).

**Table 2:** Average number of production words, PTUs and N-PTUs for Text A and Text B.

Text	Average number of production words	Average number of PTUs	Average number of N-PTUs
A	137.00	51.58	21.41
B	191.93	72.70	30.81

The average number of PTUs at each level, as well as the overall average of PTUs produced by student and semi-professional translators in the translation of Text A and Text B, were calculated and are presented in Table 3.

**Table 3:** Average number of the PTUs at each level and overall average of PTUs.

Text	Number of PTUs Participants											
		1-W^1^	2-W	3-W	4-W	5-W	6-W	7-W	8-W	9-W	≧10-W^2^	overall average
A	Students	18.83	12.70	8.54	5.61	2.89	1.73	0.94	0.47	0.34	0.49	52.81
	Semi-professionals	18.71	10.74	7.48	5.14	3.03	1.60	1.43	0.60	0.43	0.80	49.97
B	Students	25.00	16.84	12.55	7.42	3.98	2.67	1.36	0.95	0.51	0.8	72.07
	Semi-professionals	27.06	17.63	12.43	7.51	3.17	2.62	0.93	0.71	0.63	0.94	73.66

^1^1-W = 1-word PTU;

^2^10-W = ≧10-Word PTU.

**Fig 1:**
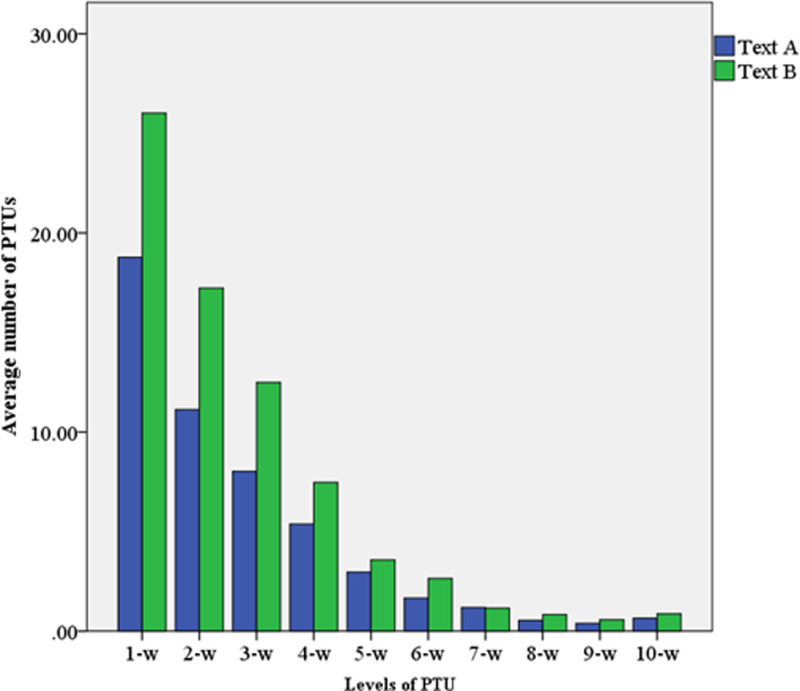
Difference in average number of PTUs at each level for Text A and Text B. 1-w = 1-word PTU; 10-w = ≥10-word PTU.

Based upon Table 3, Fig 1 was created. It is evident from Fig 1 that the number of PTUs at the 1-to-6-word levels in the translation of Text A is higher than that in Text B. Moreover, the number of PTUs at these levels decreases progressively in the translation of both texts. However, the number of PTUs at the ≥ 7-word levels, despite some fluctuations, shows no significant differences between the two texts. This observation is supported by the repeated measures ANOVA results presented in Table 4. The analysis reveals a significant main effect of ST translation difficulty on the number of PTUs at the 1-to-6-word levels (all p-values <  0.05), but no significant main effect on the number of PTUs at the ≥ 7-word levels (all p-values >  0.05). These results indicate that participants produced significantly more 1-to-6-word PTUs in translating Text B compared to Text A, whereas the number of ≥ 7-word PTUs remained unaffected. In other words, increased ST translation difficulty significantly raised the number of PTUs at the 1-to-6-word levels but had no discernible impact on the ≥ 7-word levels. Additionally, the repeated measures ANOVA results demonstrate a significant main effect of ST translation difficulty on the overall average number of PTUs, suggesting that participants produced substantially more PTUs in the translation of Text B.

**Table 4:** Differences in average number of PTUs at each level and overall average of PTUs for Text A and Text B.

PTU	1-w^1^	2-w	3-w	4-w	5-w	6-w	7-w	8-w	9-w	≧10-w^2^	overall average
F value *p* value	56.54.000	58.18.000	80.84.000	34.01.000	5.63.020	21.71.000	0.36.849	2.98.084	2.85.095	3.15.079	202.12.000

^1^1-w = 1-word PTU;

^2^10-w=≥10-word PTU.

#### Interaction effect of ST translation difficulty and translation experience on number of N-PTUs and overall average of TUs.

**Fig 2:**
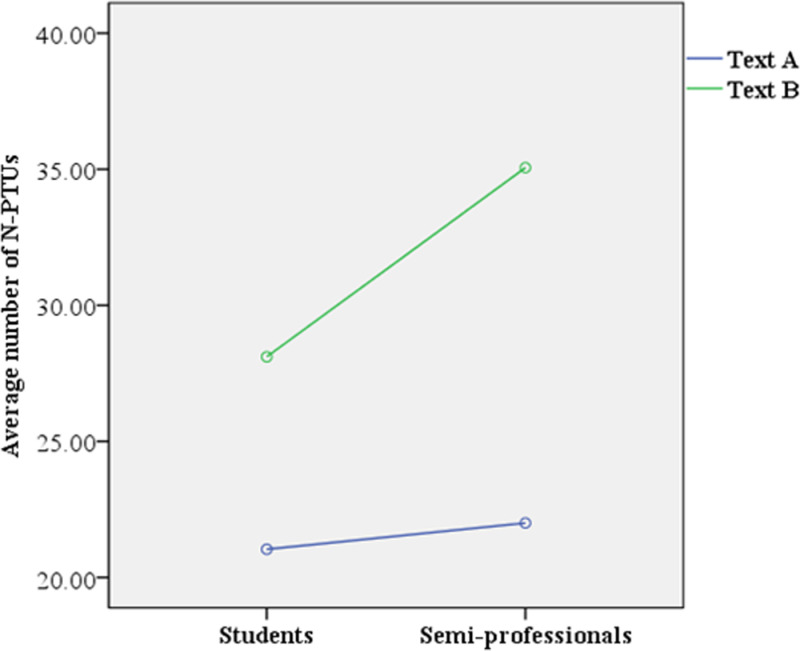
Interaction effect on average number of N-PTUs.

**Fig 3:**
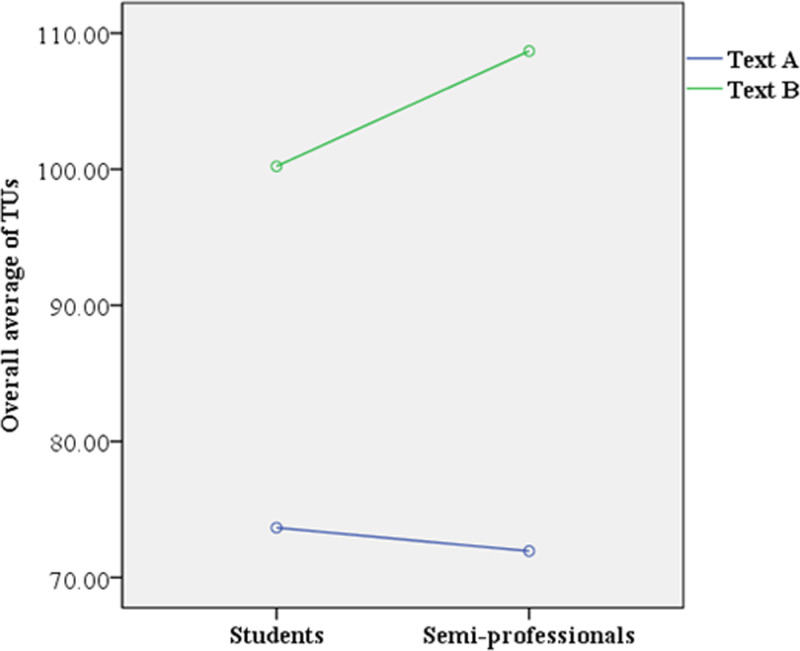
Interaction effect on overall average of TUs.

The repeated measures ANOVA results revealed a significant interaction effect between ST translation difficulty and translation experience on the number of N-PTUs (F(1, 88) =  4.974, p = .028 < .05) and overall average of TUs (F(1, 88) =  5.226, p = .025 < .05), as indicated in Figs 2 and 3, respectively. The results of the simple effects tests further exhibited that for Text A, the number of N-PTUs produced by student translators was not significantly different from that produced by semi-professional translators (F(1, 88) = .119, p = .731 > .05, Mstudents =  21.036, MSemi-professionals = 22.00). However, for Text B, student translators produced significantly fewer N-PTUs compared to semi-professional translators (F(1, 88) =  4.369, p = .039 < .05, Mstudents =  28.109, MSemi-professionals =  35.057).

Moreover, the results revealed that the overall average of TUs produced by student translators was significantly lower for Text A than for Text B (F(1, 88) =  91.512, p = .000 < .05, MText A =  73.655, MText B =  100.218). A similar pattern was observed among semi-professional translators, who also produced significantly fewer TUs for Text A than for Text B (F(1, 88) =  111.417, p = .000 < .05, MText A =  71.943, MText B =  108.686).

#### APTU size in the translation of Text A and Text B.

**Fig 4:**
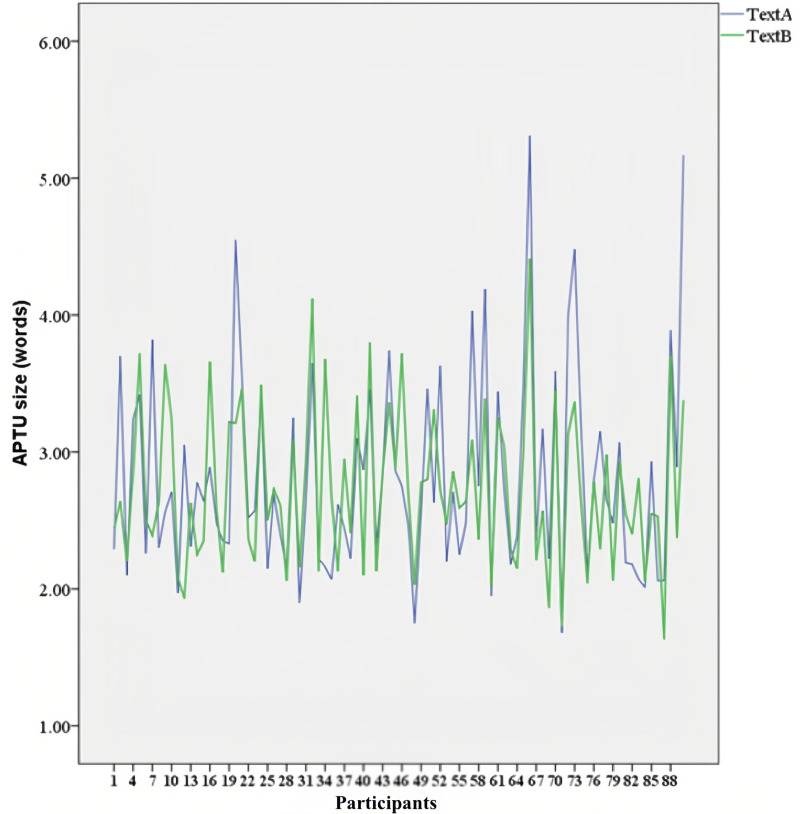
Students and semi-professionals’ APTU sizes for Text A and Text B. Participants 1–55 are students; 56–90 are semi-professionals.

The APTU sizes for each participant in the translation of Text A and Text B were calculated, and a line graph illustrating the results is presented in Fig 4. The repeated measures ANOVA results revealed a significant main effect of ST translation difficulty on APTU size (F(1, 88) =  4.221, p =  0.043 < .05). This indicates that participants employed significantly larger TUs when translating Text A compared to Text B (MText A =  2.836>  MText B =  2.735).

Despite the differences between the two texts, Fig 4 highlights considerable within-participant variation in APTU size. 20 students’ APTU sizes for Text A were slightly larger compared to Text B, while 35 students’ APTU sizes for Text A were smaller to varying degrees. Similarly, 24 semi-professional translators’ APTU sizes for Text A were larger compared to Text B, whereas 11 semi-professional translators’ APTU sizes for Text A were smaller to varying degrees. For student translators, the APTU sizes for Text A ranged from 1.75 to 4.55 words, while for Text B, the range was 1.93 to 4.12 words. For semi-professionals, the APTU sizes for Text A spanned from 1.68 to 5.31 words, and for Text B, from 1.63 to 4.41 words.

Despite these variations, the line graph in Fig 4 reveals a clear trend: the majority of participants’ APTU sizes for both texts consistently fell within the range of two to four words.

### Translation experience and number and size of TU

#### Number of PTUs produced by student and semi-professional translators.

A repeated measures ANOVA was conducted to examine the number of PTUs at each level and the total number of PTUs produced by student and semi-professional translators across both texts. As shown in Table 5, the results revealed no significant main effect of translation experience on either the number of PTUs at each level or the total number of PTUs. This finding suggests that student translators and semi-professional translators did not differ significantly in the number of PTUs produced at each level or in the total number of PTUs when translating Text A or Text B.

**Table 5:** Differences in number of PTUs at each level and total number of PTUs produced by student and semi-professional translators.

PTU	1-w^1^	2-w	3-w	4-w	5-w	6-w	7-w	8-w	9-w	≧10-w	Total^2^
*F* value	.208	.318	1.054	.229	1.332	.138	.029	.097	.610	1.383	.458
*p* value	.649	.574	.307	.633	.252	.711	.866	.756	.437	.243	.501

^1^1-w = number of 1-word PTUs;

^2^Total = total number of PTUs.

### Size of PTUs for student and semi-professional translators

#### Difference in APTU size between student and semi-professional translators.

A repeated measures ANOVA was performed to analyze the APTU sizes of student and semi-professional translators in translating Text A and Text B. The results revealed that the main effect of translation experience on APTU size was not statistically significant (F(1, 88) =  0.460, p =  0.499 >  0.05). This finding suggests that there was no significant difference in APTU sizes between student translators and semi-professional translators.

### Interaction effect of translation experience and translation difficulty on APTU size

**Fig 5:**
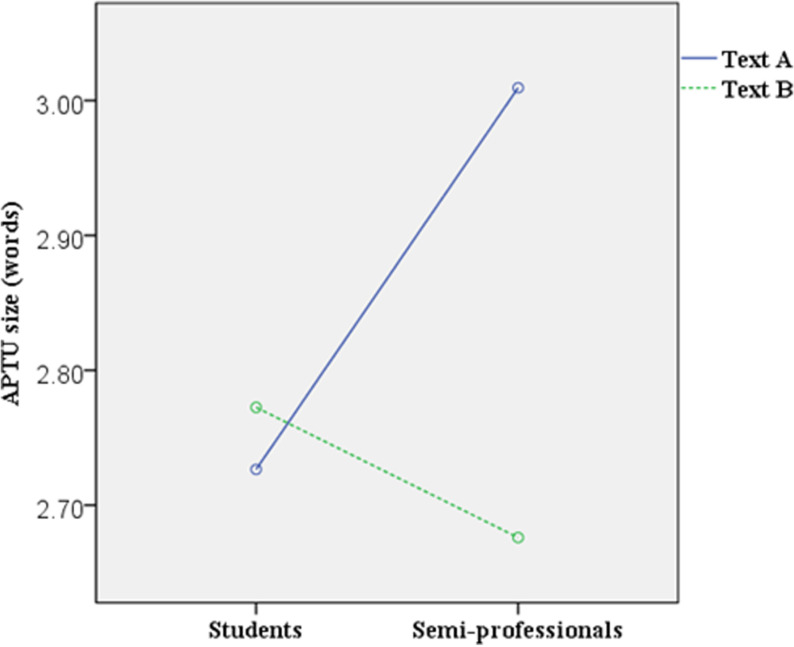
Interaction effect of translation difficulty and translation experience on APTU size.

The repeated measures ANOVA results, as illustrated in Fig 5, revealed a significant interaction effect between ST translation difficulty and translation experience on APTU size (F(1, 88) =  7.355, p =  0.008 < .05). To further investigate how APTU size varies across different combinations of translation difficulty and experience levels, simple effects tests were conducted. The results showed that for student translators, the APTU size for Text A was not significantly different from that for Text B (F(1, 88) =  0.278, p =  0.599 > .05, MText A =  2.726, MText B =  2.772). However, for semi-professional translators, the difference was significant: the APTU size for Text A was notably larger than that for Text B (F(1, 88) =  9.295, p =  0.003 < .05, MText A =  3.009, MText B =  2.735).

## Discussion

This study aims to examine how student and semi-professional translators process TUs in terms of their number and size when translating two texts of varying difficulty. The research questions have been thoroughly addressed. In the following sections, the experimental results will be discussed by revisiting each research question individually.

### Impact of translation experience on number and size of TUs

As illustrated in Fig 4, the APTU sizes for both student and semi-professional translators predominantly fall within a range of two to four words, albeit with notable variations. Specifically, the largest APTU size observed was 5.37 words, while the smallest was 1.68 words. These findings align closely with those of previous studies conducted by Lörscher [12], Krings [11], Dragsted [13], and Gerloff [18], which suggest that a prototypical TU typically comprises a group or clause containing two to six words. This discovery offers fresh evidence for determining the size of a prototypical TU. The number of linguistic chunks within a TU corresponds closely to the capacity of human working memory (WM), which has been found to range from 2 to 5.5 words [41,42].

While it is acknowledged that the exact size of TUs used by translators may vary somewhat based on their level of experience [43], this study found no significant impact of translation experience on APTU size. This finding supports the conclusions drawn by Kiraly [19] and Dragsted [13], but contrasts with the results reported by Carl and Kay [3] and Jakobsen [28]. The discrepancy between these studies may be attributed to the differences in translation direction of the tasks involved. In this study, following the methodologies of Kiraly [19] and Dragsted [13,29], participants were engaged in inverse translation (native language to foreign language). In contrast, in the studies by Jakobsen [28] and Carl and Kay [3], participants were involved in direct translation (foreign language to native language). As noted by Ferreira [44,45], translation direction exerts different levels of cognitive pressure on translators, with inverse translation demanding more cognitive effort than direct translation. Therefore, it is plausible to assume that the inverse translation task in this study imposed significant cognitive pressure on both student and semi-professional translators. This increased cognitive pressure may have consumed the majority of their cognitive resources, rendering their WM capacity insufficient to process larger TUs. Consequently, no significant difference was observed in the size of TUs between student and semi-professional translators.

To account for the lack of significant differences in the APTU size between student and semi-professional translators, it is also important to consider the cognitive processing mechanisms underlying this phenomenon, particularly through the lens of the cumulative effect of translation experience. One possible explanation is that while semi-professional translators may have more translation experience than student translators, this experience might not yet be sufficient to significantly alter their TU processing patterns. It is plausible that the effect of accumulated translation experience needs a longer period of practice and professional development before it becomes evident in the form of significantly larger APTU sizes. As noted by Sirén and Hakkarainen [46], expertise in translation develops incrementally through a process of accumulation and refinement of both linguistic and cognitive skills. The initial years of translation practice may not significantly shift cognitive patterns related to TUs, but as translators become more familiar with specific types of texts and translation tasks, their cognitive processes begin to evolve in a more noticeable way.

Indeed, a key factor here is the “threshold” effect in expertise development. For semi-professional translators, the additional experience gained through professional practice may not be enough to push them past a threshold where significant differences in cognitive processing (such as changes in the APTU size) become observable. Translation experts, as described by Remkhe and Nefedova [47], undergo a phase in which their processing patterns become more intuitive and efficient, but such changes might only be apparent after a substantial amount of accumulated experience. Therefore, it is possible that the semi-professional translators in the study are in a transitional phase, where their cognitive processing strategies have not yet reached the level of efficiency that would result in a substantial shift in size of TU.

This lack of significant difference in size of TU also explains why the number of TUs produced by students was not significantly different from that produced by semi-professional translators. If translators are translating a text using TUs of similar sizes, it stands to reason that the number of TUs they use to complete the task will not vary significantly.

### Impact of ST translation difficulty on number and size of PTUs

The results showed that the main effect of ST translation difficulty was significant in number and size of TU, with increased translation difficulty resulting in an increased number and reduced size of TUs. This finding basically echoes those of Dragsted [13,29], Buchweitz and Alves [48] and Wang [2,30] and could be accounted for within the theoretical framework of cognitive psychology.

As is known to us, translation is an information processing process supported by WM, where information can be temporarily stored and processed. Due to its limited capacity, it is difficult for the information processing system to simultaneously solve multiple tasks, and processing more difficult linguistic items consumes more WM capacity than processing less difficult ones. As Newell and Simon [49] and Campbell [50] argue, difficult items in the ST make great demand on the limited WM capacity. When a large amount of the total WM capacity is used to comprehend or produce a particular item, it must be assumed that less, or no, capacity is left to concentrate on other elements, and consequently the presence of a problematic lexical element in the ST will reduce the number of items in a TU, possibly to only one word. Therefore, it might be assumed that in this study in the translation of a more difficult text, the sizes of TU for both student and semi-professional translators naturally become smaller.

Similarly, as size of TU decreases, the translator must produce more TUs to complete the task, leading to an increase in the overall number of TUs as translation difficulty rises. However, it is important to note that this increase primarily involves TUs comprising 1–6 words, rather than those comprising ≥ 7 words, which do not significantly contribute to the higher TU count observed in more challenging texts.

### Interaction effect of translation experience and ST translation difficulty on APTU size, number of N-PTUs and overall average of TUs

#### On APTU size.

The results revealed a significant interaction effect between translation experience and ST translation difficulty on APTU size. Simple effects tests further indicated that the APTU size of semi-professional translators decreased significantly when translating the difficult text compared to the relatively easy text. However, this pattern was not observed among student translators. This finding partially aligns with that of Dragsted [13], though it differs in that the semi-professionals’ APTU size for the difficult text was unexpectedly smaller than that of the students. This anomaly may be attributed to the nature of Text B, a legal text, which likely influenced the semi-professionals’ cognitive processing [48], and their specific level of translation experience.

All the participants reported having no prior experience translating legal texts. The primary challenge lay in translating legal terms and expressions, which, as observed by Jääskeläinen and Tirkkonen-Condit [26] and Kiraly [19], cannot be translated through automatic one-to-one correspondence. Consequently, the participants had to employ problem-solving strategies and invest significantly more cognitive effort to complete the task. Although cognitive effort cannot be measured directly, it can be inferred from temporal effort, particularly the duration of pauses during task execution [51]. Longer pauses are indicative of more demanding cognitive processes [13,37]. In both translation and post-editing, pauses have been recognized as markers of cognitive effort [29,52–55], consistent with findings in monolingual language production [34,35]. To determine whether the participants expended more cognitive effort in translating Text B, pause durations were calculated for both texts across the two translator groups, and a repeated measures ANOVA was conducted. The analysis revealed a significant interaction effect between translation experience and translation difficulty on pause duration (F(1, 88) =  4.105, p = .046 < .05). The simple effects tests results further showed that semi-professional translators’ pause duration for Text B was marginally longer than that of student translators (F(1, 88) =  3.209, p = .077 > .05, MSemi-professionals =  1464.346>  MStudents =  1254.908). This suggests that semi-professional translators invested relatively more cognitive effort in handling the challenges posed by the legal terms and expressions in Text B.

Apart from the type of the ST, another factor that may partly explain the semi-professional translators’ smaller APTU size for Text B is their level of translation experience. While they have improved their theoretical knowledge through courses on translation theory and strengthened their translation skills through practice and fieldwork, they remain, at their core, translation learners. As Bernardini [56] noted, semi-professional translators, despite developing some awareness of translation problems, have not yet achieved the level of automaticity in problem-solving seen in professional translators. Similarly, Göpferich [57] argued that unfamiliar text types, combined with heightened translation problem awareness, demand greater working memory capacity for problem-solving. Therefore, it may be safe to assume, when translating the more challenging text, the semi-professional translators’ increased cognitive effort and problem awareness may have consumed a substantial portion of their working memory capacity, limiting their ability to process larger PTUs.

As to the student translators, when translating Text B, they also need to process legal language and terminology, which inherently demands greater cognitive effort to address translation challenges. However, due to their limited translation practice and lack of experience, they may have not fully developed a high level of translation problem awareness. As Bernardini [56] points out, experienced translators tend to have a heightened greater awareness of translation problems compared to novice translators. With their accumulated experience, semi-professional translators are more adept at identifying and resolving translation difficulties, whereas student translators, due to their lack of experience, are often less sensitive to certain translation challenges. Consequently, the student translators might have failed to fully recognize the challenges posed by legal terms and expressions in Text B. Their lower sensitivity to ST translation difficulty results in a reduced cognitive strain compared to semi-professional translators, as evidenced by their relatively shorter pauses when encountering translation challenges. The reduced cognitive effort and lower problem awareness does not place significant pressure on the working memory of student translators. As a result, the student translators tend to maintain relatively larger TUs in translating Text B, as they do not consciously break the text down into smaller cognitive segments for problem-solving.

#### On number of N-PTUs and overall average of PTUs.

The results revealed that semi-professional translators produced significantly more PTUs and N-PTUs for Text B than student translators. According to the definitions of PTU and N-PTU, generating more TUs in the translation of Text B indicates greater segmentation in the TT production process, while producing more N-PTUs suggests increased recursiveness in the translation process. N-PTUs generally lack a direct equivalent linguistic entity. Their constituents—such as backspaces, deletions, spaces, cursor movements, carriage returns, and mouse navigations, or combinations of these—serve as markers of translation recursiveness [48,58–60]. In Translog terms, these actions correspond to what are referred to as “revision keystrokes” [48], and a higher frequency of such strokes signifies greater recursiveness.

More recursiveness (more N-PTUs) combined with higher segmentation (more PTUs) can be an indication of translator’s adaptive behavior to task difficulty. If there is no increase in recursiveness, but merely in segmentation, the translator may be simply caught in a trap he or she does not have the strategies to get out of. If, however, there is increase in segmentation and in recursiveness, arguably, there is an adaptive response to increased task difficulty: the translator is trying to claw his or her way out of a more difficult rendering [48]. Given that the semi-professional translators produced more N-PTUs and PTUs when translating Text B, it can be inferred that they made significant efforts to adapt to the increased difficulty posed by the legal terms and expressions in Text B.

## Conclusion

This study investigates the processing of TUs by student and semi-professional translators based on keylogging data. Two texts of varying difficulty—one relatively easy and one more challenging—were used to analyze the number and size of TUs. The findings indicate no significant differences in the number of PTUs or the APTU size between student and semi-professional translators. However, notable differences emerged between the two texts: for the more difficult text, the APTU size was significantly smaller, the number of 1–6-word PTUs increased substantially, while the number of ≥ 7-word PTUs remained relatively unchanged. Additionally, when translating the difficult text, semi-professional translators, compared to student translators, demonstrated smaller APTU sizes, a higher number of N-PTUs, and a greater overall average number of PTUs. These findings shed light on the intricate interplay between cognitive effort, ST translation difficulty, and translation experience in TU processing.

The findings of this study have some implications for both design of translation courses in academic settings and development of professional training programs for translators. First, the significant impact of ST translation difficulty on TU processing underscores the need for translation curricula to include texts of varying levels of translation difficulty. For translation learners more challenging texts should be gradually introduced to enhance problem-solving skills and resilience under cognitive strain. Second, the study highlights differences in size and number of TUs based on ST translation difficulty, particularly the increased reliance on smaller and more numerous TUs when handling difficult texts. Exercises such as segmenting sentences into smaller chunks and practicing re-structuring sentences can help students adapt to the demands of different STs. Third, the finding that semi-professional translators produced a higher number of N-PTUs when translating difficult texts suggests that experienced translators actively engage in iterative processes, such as revising, deleting, and navigating within the text. These behaviors reflect their problem-solving and quality assurance strategies. In translation courses, students should be trained to view these non-production actions not as errors but as integral steps in refining their output. Simulation-based training, where learners perform real-time corrections using keylogging tools, could be effective in fostering such practices.

However, several limitations should be acknowledged. First, it involves a single language pair (Chinese-English), which may limit the generalizability of the findings to other language pairs. Second, the study adopts a cross-sectional design, which provides a snapshot of TU processing but does not capture longitudinal developments in translators’ cognitive processes. Third, the use of keylogging as the sole method of data collection might overlook other important cognitive and contextual factors influencing TU processing, such as eye-tracking data or TAPs.

To address these limitations, future research could adopt a longitudinal approach to examine how TU processing evolves with translation experience over time. Investigating the development of TU processing across different stages of expertise, from novice to professional translators, would provide deeper insights into cognitive adaptations in translation. Additionally, extending the study to include diverse language pairs could reveal how language-specific features influence TU categorization and processing. Finally, triangulation of data, such as combining keylogging with eye-tracking or TAPs, would offer a more comprehensive understanding of the interplay between cognitive effort and TU processing in translation.

## Supporting information

S1 File Data on translation experience, translation difficulty, pausing & drafting time, number and size of TUs, etc. (XLSX)
